# Actors involved in the regulation of clinical research: comparison of Finland to England, Canada, and the USA

**DOI:** 10.1186/s12961-015-0009-8

**Published:** 2015-04-07

**Authors:** Elina Hemminki

**Affiliations:** National Institute for Health and Welfare, P.O. Box 30, 00271 Helsinki, Finland; Department of Public Health, University of Helsinki, Helsinki, Finland

**Keywords:** Comparative study, Drug control, Governance, Research regulation

## Abstract

**Background:**

The relevance and quantity of clinical research has caused concern and regulation is claimed to hinder clinical research. This paper compares clinical research regulations in Finland to those of England, Canada, and the USA around 2010–2011.

**Methods:**

Several approaches and data sources were used, including semi- or unstructured interviews of experts. For the analysis, a theoretical framework was made, data from various sources was synthesized, and features of the systems were simplified and classified. The various specific names and terms used in the data were changed into general ones.

**Results:**

Common structures for the regulation existed in all four countries, but the details and scope varied. The research regulated within the main system was determined by research type (Finland), the financer of the health system (England), or research site (Canada, USA). Only Finland had specific legislation on medical research. The overriding impression of the regulatory systems was one of complexity. All countries had extra regulation for drug research. The types of drug research covered varied from trials with unlicensed (new) products or new indications (USA and Canada), to all types of interventional drug research (England), where ‘interventional’ was interpreted broadly (Finland). The complexity of regulations had led to the creation of various big and small businesses to help researchers and sponsors. There was notable variation in the role played by the public research funder. The role played by health care was difficult to study and seemed to involve varying interests as researchers were also health care employees. Research ethics committees were important and their tasks also included aspects other than ethics.

**Conclusions:**

This study revealed that a comparison between countries can provide useful insights into the distinctive aspects of each country’s system, as well as identifying common features that require international action.

## Background

The regulation of clinical research is governed by two opposing sets of interests. While demand for evidence-based medicine and the interests of health care and industry favor research facilitation, the regulators, patient protection, and autonomy are putting the case for greater regulation. Concern has been raised about the relevance and quantity of clinical research and it is even claimed that regulation hinders research activity [1-4]. Due to the high volume of drug trials, strong commercial interests, tighter control traditionally, and problems related to the European Union (EU) Clinical Trials Directive, a special focus has been placed on drug research.

The aim of this paper is to compare clinical research regulation in Finland to that of three other countries, in order to illustrate common features and variations around the period 2010–2011, while focusing on the various actors involved in research regulation. Research ethics committees (RECs) will be discussed in more detail in another paper (Hemminki, unpublished manuscript 2015); only their general structures are presented here. In all countries compared, particularly England, changes have occurred since 2011, but in order to preserve a common timeframe these changes are referred to only briefly. Regulation concerns both research ethics (content) and practical issues (research governance); in this article, the term research ‘regulation’ is used to cover both. The focus is on clinical research, defined as medical research conducted on and with patients. Regulation of medical research using animals, body parts (samples), or information alone, including the regulation of registers, records, and bio-banks is not covered; privacy and data protection issues are not independently considered.

Previous studies have investigated research regulation in Finland [1,2,5], highlighting various elements in need of improvement. To better understand the regulation system and to get ideas for improvements, comparisons were made with three other countries. The comparison countries were chosen based on the assumption that they have a great interest in research regulation, as well as for reasons of convenience (contacts through which data collection could be organized). Finland is an EU country with largely publicly funded health care, conducting much clinical research for its size. England (UK) is an EU country with a national health care system, a great deal of clinical research and ongoing discussion of research regulation. Canada has publicly funded health care. To some extent, research regulation varies by province and our results are mainly based on Ontario, Canada’s largest province. The USA, a leader in medical research, has a major international impact on the field.

## Methods

Several approaches and data sources were used. The key method applied consisted of semi-structured and unstructured interviews of experts. In addition, use was made of previous reports, documents, presentations in meetings, observations and, in the case of Finland, previous knowledge. Empirical data was collected: in Finland in 2009–2011, in Canada in late 2010, in England in early 2011, and in the USA in late 2011. Some data was completed up to spring 2014 using web pages and publications and, later, interviews.

The data collection conducted in Finland has been reported in more detail in previous publications [1,2,5]. A total of 26 experts involved in Finland’s research and health services were purposefully selected to cover the expertise of clinical research policy makers and regulators. All of the experts approached, or their substitutes, agreed to be interviewed (15 face-to-face and 11 via telephone) by two researchers with a medical background; 22 chairpersons from 25 official medical RECs were interviewed by two other researchers, a dentist and a lawyer.

In the comparison countries, the persons interviewed were chosen based on previous knowledge of important institutes in this context, suggestions made by the interviewees, geographical proximity, and availability during the data collection visits. All persons contacted agreed to participate if the timing was possible. Interviews were performed by a researcher with a medical background. In England (London, Oxford), 21 persons were interviewed in spring 2011; in addition, various informal discussions were held with experts. Feedback interviews were conducted with four interviewees in late 2013. An important source of data was the report and background documents on health research regulation [6]. In Canada (Toronto), 13 persons were interviewed in the fall of 2010; in addition, informal discussions were held. Six seminars relating to research regulation were attended and a meeting of a hospital ethics committee was observed. In the USA (Washington, DC and Baltimore), 24 persons were interviewed and various informal discussions were held in the fall of 2011. Four seminars relating to the regulation of clinical research were attended. Informal discussions on the study findings were held with a few experts in England and the USA in the fall of 2013. In spring 2014, a draft report was shown to two persons in Canada, England, and the USA, most of whom had previously been interviewed. The report was modified in accordance with their comments.

Most interviews were semi-structured, but some were unstructured and resembled normal discussions. The themes of the interviews and some pre-prepared questions were drawn up prior to each interview, but the actual interview and its focus varied in accordance with the expert’s position, experience, and emergent information. Information and material from previous interviews were utilized in subsequent interviews. In Finland, the original questions and themes were chosen by previous literature and the project researchers’ knowledge and experience of research regulation; the questions were reformulated when new information from the interviews accumulated. In the comparison countries, the interviewees were approached with an open mind. Data collection was a learning process: the questions and items were first prepared according to the Finnish experience and background reading, and reformulated by new information in subsequent interviews.

The interviews lasted from 30 minutes to 3 hours. They were not tape-recorded, but notes were made and a summary of the interview was drawn up immediately afterwards. Although a few interviewees were reserved to begin with, with the exception of two, they soon relaxed, a friendly atmosphere prevailed, and the interviewees opened up to share their expertise.

In each country, documents were collected from the web pages of the institutions involved, or were handed over during the interviews. The relevant publications were sought from literature databases and from references given during the interviews.

The analysis was material based (grounded theory). During data collection, preliminary classes of regulation items were raised and noted down. Following data collection, tables, organized by regulation dimension each containing many items, were created. The tables were preliminarily filled, as recalled from the data collection. Then, country-specific interview notes and documents were iteratively read by one researcher; notes were made on various topics and dimensions using self-adhesive notes, and organized by topic/dimension and by country. The framework was modified and made more detailed during note making. Data from various sources was synthesized and the features of the systems were simplified and classified. If an item was not found in the original country-specific notes, documents and web-pages were searched for if the item was an unequivocal fact.

### Permissions and ethics

A positive statement was issued by the THL (National Institute for Health and Welfare) ethics committee on the project as a whole (MERGO ethical review and administrative governance of clinical research) (June 17, 2010, amendment Jan 27, 2011). All interviews were voluntary; the interviewees understood the purpose of the interviews and were interviewed as experts. In Finland, the main project was explained and the purpose of the interview (to obtain an expert view) was stated. In the comparison countries, the main project was described only superficially, but the purpose of the visit and the interview (to obtain comparative data and gain a better understanding of the principles of research regulation) was explained. Personal views and experiences were requested as well as facts. The interviewees were reassured that their names would be kept confidential. The documents used were public.

### Terms used

Different terms, varying between the interviewees and documents, were used in each country. Specific names and terms were transformed into general ones, of which the key terms are: clinical research, research conducted on and with patients; clinical trial, research that evaluates interventions using experimental methods (not restricted to drugs); health ministry, the ministry/department/government office which deals with health issues (usually in addition to other matters), with the name and organizational structure varying by country; research ethics include human subject protection, conflicts of interest, and research integrity (avoidance of data falsification, etc.); RECs, known as research ethics boards in Canada and institutional review boards in the USA; ‘regulation’ covers laws, rules, overseeing, and governance.

## Results

### Context

Finland is a Nordic state in the EU, with a population of around 5 million (Table 1). Its health services have two dominant systems: a municipality-based, tax-funded, area-based system covering most in-patient care and much outpatient care, and a national health insurance scheme subsidizing part of private care, occupational care, drugs, travel, and the costs of sickness absence from work. England is an EU country with a national health service – an area-based, tax-funded, government-led system. In early 2011, outpatient and hospital care were administered and financed by trusts. The system has recently undergone various changes.

**Table 1:** **Context of clinical research regulation in Finland, compared to England, Canada (Ontario), and the USA around 2010**

	**Finland**	**England**	**Canada**	**USA**
Population size (millions)	5.4	56	13	312
Health services	Mainly publicly funded and organized	Publicly funded and organized	Publicly funded, privately organized	Mainly privately funded and organized
Local drug industry and exports	0	+++	+	+++
Local drug industry research	+	+++	+	+++
Clinical research activity	++	+++	++	+++
Number of drug trials (relative to population size)	++	++	++	++
Government promotion of clinical research	0	+++	+	+
Government promotion of commercial clinical research	0	+	++	++
Research within same regulations	Medical research narrowly	Medical research broadly	Broad (human research)	Broad (human research)
Specific laws on drug trials	Yes + EU directive	Yes + EU directive	Yes	Yes

0/+ provides a subjective estimate of the importance of the issue, relative within the four countries; 0, no or little activity.

EU, European Union.

In Canada, health care providers were privately organized but publicly funded via a detailed billing system. Health care facilities were owned by non-profit corporations. Units and practitioners billing the government were not allowed to run a private practice, making private practice marginal. The USA had a patchwork of different systems. Health care was largely privately organized and private insurance systems played an important role. However, private services obtained a notable part of their income from tax-paid insurance (Medicare and Medicaid) and there were also large federal and state-organized services. Most health care providers worked without a defined population base, although there were also large pre-payment systems.

In Finland and Canada, the local drug industry and its role in research were small (Table 1), but many small bio-technology companies and other health technology firms operated. In England and the USA, the drug industry was strong, with many large multinational companies. There were also many health technology firms of other kinds.

All four countries had a strong tradition of clinical research (Table 1). The number of drug trials (in relation to the population) was high in all four countries. Practically all clinical research was carried out through health services, but some research institutes also performed clinical research. Phase I drug trials (done among healthy volunteers) sometimes occurred in special research centers.

### Government facilitation of clinical research

In Finland, the health ministry distributed state subsidies for university level research on public health services, but they have been notably declining in size (Table 1). The subsidies were paid to municipalities as compensation for research costs and were not regarded as facilitating clinical research as such. In England, the National Health Service (NHS) had actively facilitated clinical research using various methods, including support via the National Institute of Health Research (NIHR). The NIHR had been an important actor through its infrastructure, funding, and clinical research networks. In Canada, various government task forces and programs had aimed to facilitate health research, but not as systematically as in England. In the USA, the government was using various funding mechanisms for health research, including the National Institutes of Health (NIH) intramural and extramural funding, the Agency for Healthcare Research and Quality, the Patient-Centered Outcomes Research Institute, and other federal agencies.

With the exception of Finland, governments had also actively promoted commercial clinical research (Table 1). The interviews and documents consistently showed that drug trial operations (and other research on health technology products) departing to lower-cost, less-regulated countries, was a driving force behind government actions. Depending on the government structure, the relative role of ministries (health, research/innovation, finance, and industry/trade) varied, as did the mechanisms of promotion.

### Scope of research within same regulations

Only Finland had detailed legislation on medical research; the USA had an enabling research law (National Research Act) (Table 1). Canada and England did not have a specific law on research or medical research, but semi-legal circulars and standing orders were issued by their health ministries. Various laws dealing with specific types of research or populations were present in all four countries. All countries had enacted a law on clinical drug trials, which varied in scope (Table 1).

In Finland, the law provided a narrow definition of medical research and other health research, and other human research had voluntary regulations. In England, there were separate regulations governing medical and other human research, but the definition of medical research was broad. In Canada and the USA, regulation of medical and clinical research was part of human research regulation, covering all research involving humans or body parts. With the exception of Finland, the rules varied based on where the research was being performed or who was funding it (see the section on RECs).

### National leadership

In Finland, clinical research was not the identified responsibility of any ministry; the tasks were mainly divided between the health and education ministries (Table 2). The medical research law was initiated and sub-laws issued by health ministry, but the ministry was not actively involved in clinical research. The education ministry was responsible for academic research in general. Regulatory tasks were divided up, with ethics committees (under health services) and a drug regulatory agency (under the health ministry) being the strongest actors.

**Table 2:** **Actors in the regulation of clinical research in Finland, compared to England, Canada (Ontario), and USA, around 2010**

	**Finland**	**England**	**Canada**	**USA**
Responsible ministry	Two (health and education)	Health	Health	Health
National leadership	None	NHS ^a^, MHRA	CIHR	OHRP and FDA
Government research funders	0	+	+++	+++
Health care	+	++	++	+++?
Permission giver	“Head physician”/Health care unit ^b^	NHS Trust	“Head physician”/Health care unit ^b^	“Head physician”/Health care unit ^b^
Procedures for permission	Light	Bureaucratic	Effective/professional	Effective/professional
Conflict of interest bodies	No	Integrated to RECs	Yes +	Yes +++
Researchers’ impact	+	+++	+	+++
Regulation business	0	NK	++	+++
*Drug research particularly*				
Drug control agency ^c^	++	++	+	+++
Drug agency costs	Mainly fees	Mainly fees	Taxation and fees (~50%)	Taxation and fees (~50%)
Products covered	Drugs	Drugs (devices)	New ^c^ drugs + devices	New ^c^ drugs + devices + food

0/+ gives a subjective estimate of the importance of the actor, in relative terms within the four countries; 0, no or little activity. CIHR, Canadian Institutes of Health Research (a research funder); FDA, Food and Drugs Administration; NHS, National Health Service; MHRA, Medicines and Healthcare Products Regulatory Agency; NK, Not known to me; OHRP, Office of Human Research Protection.

^a^ Since 2012, the Health Research Authority has played a central role.

^b^ This task was delegated to someone among the health care providers, e.g., a clinic head.

^c^ For new drug trials.

In England, the health ministry (via the NHS) had a strong position. Several bodies within the NHS were involved in regulating clinical research. The Health Research Authority (HRA), established at the end of 2011, had started to play an important role. The drug and device control agency (Medicines and Healthcare Products Regulatory Agency; MHRA) regulated clinical research using drugs and medical devices. In Canada, the health ministry was the responsible ministry, acting through the funding requirements of the Canadian Institutes of Health Research (CIHR). However, the CIHR was a component of the Tri-Council funding agency, working under the same rules as other government research funders which were under other ministers. In the USA, the health ministry (Department of Health and Human Services, DHHS) was the leading ministry in clinical research regulation. Its office of research regulation (Office of Human Research Protection; OHRP) played a coordinating role in regard to other departments and agencies, and interpreted federal research regulation (Common Rule). Another DHHS agency, the Food and Drug Administration (FDA), regulated trials on new drugs and devices; FDA functions had had spill-over effects on other types of clinical research and other regulators using drug research rules for other type of research.

### Government research funders

In Finland, government research funders, including the Academy of Finland, TEKES (Finnish Funding Agency for Technology and Innovation), and the health ministry (subsidies for health services), had not been important in regulating clinical research (Table 2). In England, both the Medical Research Council and private research funders had issued guidance on clinical research. However, they had played a more modest role than the NHS. In Canada, guidelines by the main government funders – including the CIHR – on ethics and related procedures formed the cornerstone of clinical research regulation. These guidelines, the Tri-council policy statement, covered all types of research involving humans. They were enforced by requiring that all research conducted within an institute abide by them, the institute to be eligible for any funds from the CIHR or other government funders. In the USA, to be eligible for federal funding, either for research or health care, an institute had to follow the rules issued by the relevant ministry (DHHS). Furthermore, the NIH had issued extensive regulations covering intramural research, with spill-over effects on extramural funding.

### Health care organizers and providers

In all four countries, permissions for clinical research projects were granted by a nominated person at the sites at which patients were recruited or data collected; if external funding was being used, contracts were also usually made (Table 2). Health care units also hosted RECs. Institutions, rather than individual researchers, bore the relevant legal and financial responsibility, for which reason various mechanisms had been created to avoid ethical and legal problems in research. In the USA and England in particular, it was reported that real and alleged scandals had raised health care providers’ interest in this.

On the other hand, university level institutes wanted to promote research; there was concern that commercial research, in particular, might migrate from university hospitals to more attractive sites (smaller hospitals, primary care, private research companies) and countries. In all four countries, institutional structures were in place within university attached health care units, in support of researchers. In England, NHS trust offices facilitated clinical research by providing infrastructure for NHS-supported non-commercial research. In Finland and Canada, regulation and research facilitation were often intermingled at personal level, same persons being responsible both for research facilitation and control. Particularly in the USA, in university affiliated units, research was important to improving services as well as to patient and physician recruitment.

In Finland, the granting of permission for research was usually straightforward, but the paperwork could be tedious. Ethics committees also carried out administrative work and checked that laws were being followed. In England, permission to do research was granted by the care organizers, the NHS trusts. The official granting permission could be an administrator with no direct interest in having research done within the unit. Permission-seeking was not streamlined and criticism was directed at the slowness and complexity of the process. Some trusts would review the ethics and scientific value of a project and might implement their own requirements.

In Canada, the processes involved in obtaining research permission and making contracts were well organized, focusing on practical issues. The regulation structure could be divided into specific units, such as contracts and money, ethics committees, and conflicts of interest. Research was mainly administered by research institutes and administratively separated from patient care and hospitals. Due to the complexity of the system in the USA, a full picture of the interests of health care organizers and providers was not obtained. However, RECs also seemed to play a key role with regard to permissions, contracts, and other practical issues, even though these were administratively separate.

### Conflicts of interests (COI)

COI regulation was mainly understood to involve financial issues. In Finland, there were no separate bodies for checking COI, a vague duty of RECs (Table 2). No transparency among research participants was required, and no structures existed for overseeing competition or other conflicts of interest between the permission-giver and the researcher. In England, RECs were given the task of checking researchers’ COIs and National Research Ethics Service (NRES) advised that research participants should be informed about COI. In Canada and the USA, specific bodies or designated persons were assigned to check on COI issues. In Canada, checking was performed by local research offices, but the transfer of such duties to special boards was under planning. In most cases in the USA, special offices or nominated boards reviewed COI issues and disclosers.

### Academic actors

Scientific journals and associations, researchers, universities, and other scientific bodies had had a major impact on clinical research regulation by creating rules and practices, through education and self-regulation, and through researchers becoming research administrators. Data on academic actors were not systematically collected, but some observations from interviews and documents are given below. In all four countries, academic clinical researchers stated that they were unhappy with at least some aspects of research regulation. In most cases, the researchers had made no personal protests on this basis, since this may have harmed their (future) research projects. In Finland, clinical researchers were organized into various associations with no common voice. Unlike the drug industry, for example, they were not collectively consulted on research regulation.

In England, on the initiative of researchers, the health ministry had asked the Academy of Medical Sciences, which represents research oriented physicians, to review the system of medical research regulation [6]. This had contributed to the reorganization of NHS research regulation and the creation of the HRA. In the EU, some clinical associations, such as the European Association for Cancer Research, had actively lobbied for the improvement of EU clinical trials regulation. In the USA, a group of concerned researchers was behind the initiative to change the leading document on human research regulation (Common Rule). The Institute of Medicine had held seminars and published books on hindrances to medical research and the actions needed, with a focus on drug research.

### Regulation businesses

Regulatory requirements had led to the creation of various kinds of businesses to help researchers and research sponsors fulfill the requirements, particularly in the USA and Canada. This was less common, or at least less visible, in Finland and England. These businesses included private RECs and contract research organizations (covering the full package of regulations or specializing in certain aspects only, such as monitoring, contracts, or ethics education) and firms accrediting human research protection programs and RECs.

In the USA, public institutes and professional associations had also created programs around medical research regulation, including legal and ethical education, and trial registration. Institutions, such as the NIH, and large universities and affiliated hospitals had constructed infrastructure and hired special experts such as protocol writers, contract specialists, and patient recruitment offices. Professional ethics and regulation associations, trade unions of sorts, had been established, such as the Public Responsibility in Medicine and Research for regulators and REC members in the USA, which was running a program certifying REC members. Ethics had been professionalized to a large degree.

### Drug control agencies

In all four countries, (some) clinical research involving drugs were governed by additional laws and requirements, and had separate drug control agencies. Such agencies covered drugs only (Finland), drugs and devices (England, Canada), or drugs, devices, and food (USA). In Finland and England, the agencies were financially dependent on their tasks, with various permission fees, particularly drug licensing fees, making up most of their income. In England, clinical trial review fees were estimated to account for around 4% of the agencies’ income. In the USA and Canada, no specific charges were invoiced for trial evaluations, since these were rolled into licensing fees covering around half of the agency’s costs (Table 2). The EU’s clinical trials directive (Directive on Clinical Trials on Medicinal Products in Human Subjects, CTD, 2001/20/EC) was a supranational law regulating drug research in Finland and England. It had been criticized in various respects; it was claimed that it had discouraged non-commercial research and made research costly and bureaucratic.

The types of drug research covered varied (Table 2). In Canada and the USA, only trials with unlicensed (new) drugs or those on new indications (or administration increasing risks) were covered. The EU’s clinical trials directive stipulated coverage of all ‘interventional’ drug research. In Finland, the Finnish Medicines Agency (FIMEA) interpreted the directive as even applying to some surveys and other non-experimental drug research. However, the FIMEA had simpler procedures for research on licensed drugs and waivers of fees in cases of non-commercial researchers. In England, the MHRA had worked to narrow the interpretation of the EU directive in regard to licensed drugs and suggested a proportionate review, i.e., tailored to fit the expected risk.

The clinical trials directive had had an impact on clinical research regulation over and above drug trials. In Finland, the directive was integrated into the medical research law, influencing the principles governing other types of medical research. In England, the directive requirement for one-committee handling of multi-center studies had also led to one-committee handling in other medical research.

Because the agencies reviewed drug research independently of RECs and research permission givers, drug trials were reviewed twice (or three times). In Finland, the review focus in the drug agency was on legal and safety aspects, reflecting the main tasks of the drug agency (licensing medicines for sales, inspecting producers and sellers, and overseeing marketing). Physical inspections of trial sites were rare. A problem was the lack of scientific resources for reviewing novel research. In England, trial site inspections played a larger role than in Finland. The way in which the inspections were performed was criticized as often intimidating researchers.

In the USA, the drug control agency (FDA) had traditionally played a strong role in overseeing drug trials. Trial reviews were integrated into licensing procedures, and the same persons performed both reviews. Review was thorough, and selective onsite inspections of actors involved in drug trials – including researchers and RECs – were important. The law was highly specific with regard to trial contents and the FDA had been proactive in setting standards. However, advice and regulation were concerned only with research for drug licensing. The agency advised researchers or their sponsors on how the agency thought a trial should be performed to make it useful for licensing. An educational approach of this kind seemed acceptable to the drug companies, but not always to established academic researchers. In Canada, according to interviewees, the drug control agency (Health Canada) played only a modest role in drug trial regulation. The agency inspected a small sample of the trials underway.

### Research ethics committees (RECs)

In all countries, RECs were key actors in regulating clinical research (Table 3). Their functions involved more than just evaluating the ‘ethics’ (human subject protection) of individual projects. Their main structural features are described here and a more detailed description of their work will be given in another paper (Hemminki, unpublished manuscript 2015).

**Table 3:** **Research ethics committees (RECs) in clinical research regulation** ^**a**^ **in Finland, compared to England, Canada (Ontario), and USA, around 2010**

	**Finland**	**England**	**Canada**	**USA**
Importance	+++	++	+++	+++
Main basis	Research law; EU	Health ministry + NHS regulation; EU	Requirement from research funder CIHR	Research law; health ministry regulation
Area responsibility	Yes	No, inclusive	No, selective	No
Number	Few, law defined	Declining, NRES defined	Many, free	Many, free
Private RECs	No	Few	Yes	Yes, important
All clinical research in the main system	Yes	Not research outside NHS	Not research outside health care units	Yes
Appointing body	Hospital district	NRES	Hospital board ^b^	Hospital ^b^/private
Funding	Fees + hospital district	NHS	Fees + hospital ^b^/grants	Hospital ^b^/fees (private)
REC control body	No	Yes, strong NRES	No	Yes, OHRP + FDA

0/+ provides a subjective estimate of the importance of the issue, on relative terms within the four countries; 0, no or little activity. CIHR, Canadian Institutes of Health Research; EU, EU Clinical trials directive; FDA, Food and Drug Administration; NRES, National Research Ethics Service; NHS, National Health Service; OHRP, Office of Human Research Protection.

^a^ Within the main health care system; two systems in the USA.

^b^ Hospital or other health care unit.

In Finland, the medical research law required RECs to deal with ‘medical research’, and the law and its sub-laws were detailed in RECs mandate and tasks. In England, RECs for NHS-based research were specified in regulations issued by the health ministry. RECs did not constitute legal bodies in general, but functioned as such under separate laws, regardless of the location of the research, in regard to specific patient-groups (such as mentally handicapped persons) and interventions including drugs. In Canada, RECs had been formalized under the requirements of the main public funder. In the USA, the national research act stipulated that human research had to be approved by a REC. More detailed requirements were stipulated under federal government regulations (Common Rule).

In Finland, RECs were area-based and had a monopoly: they handled all medical research (as defined by law) in their area (determined by the location of the principle investigator) (Table 3). In England, researchers could choose an NHS REC, which then had to consider the proposal. In Canada, RECs were health facility (hospital) based, substance based, or private. If the place from which the patients came had a REC, researchers were supposed to use it. RECs could choose which applications they handled. In the USA, the situation was the same as in Canada, but private committees played a bigger role.

In Finland, only a small and regulated number of RECs dealt with clinical research (21 (5 since 2010) and a central committee) (Table 3). In England, since 2004, the number of RECs had been regulated by the REC control body and had declined from around 200 in 2002 to around 80 in 2010 (69 in 2013). In Canada, RECs could be freely established and were numerous, and practically all university-affiliated hospitals had their own. Likewise, the USA had a large number of RECs (around 4,500), and several RECs could be located within one institute or hospital.

In Finland, there were no private RECs (Table 3). In England, only 5 private RECs remained by 2010, which mainly handled applications for Phase I drug trials. Private RECs were regulated by a government body (Appointing Authority for Phase I Ethics Committees) and funded by handling fees and a health ministry grant. These committees were amalgamated with the NHS system by 2013. In Canada, private RECs were used by commercial firms, particularly for Phase I drug trials on special testing sites, and by small hospitals/health facilities which did not have their own RECs. Private RECs could not be used if the research site was a receipt of government research funding. Private RECs were unregulated and attempts to create an accreditation system had not succeeded by 2010.

In the USA, private RECs played a major role. Their numbers were small (around 30), but they were large. They were much used by drug firms and other private companies, but also by large medical institutes. Most new drug trials were evaluated by private RECs. Large private RECs also had research-related activities other than ethics reviews. Large private RECs were accredited and, like other RECs, overseen by the REC control body. The drug agency (the FDA) inspected private RECs, which evaluated new drug or device trials. It could demand corrective measures or sanction RECs.

Interviewees’ opinions of private RECs varied notably. The potential problem of giving a positive statement due to incentives was obvious and ‘buying reviews’ was heavily criticized by some. Private RECs also paid their experts for reviews performed. On the other hand, private REC’s were liked by commercial sponsors and the FDA, possibly due to the speed and technical quality of their work. Furthermore, some claimed that private RECs were more independent than institutional ones, which may take account of institutional interests, and in various documents the word ‘independent’ rather than ‘private’ was used.

All four countries had RECs other than those described above, which handled health research beyond the mandate of the main REC system (Table 3). These were institutionally based and did not usually handle clinical research. In England, clinical research performed outside the NHS, for example abroad, could be handled by research institute RECs.

In Finland, official RECs were appointed by hospital districts (formally only since 2010) (Table 3). In England, since its creation in 2000 the REC control body had appointed and advertised local REC members; before then, RECs had been appointed by NHS trusts. In Canada, institutional RECs were appointed by hospital/health facility boards. In the USA, appointment procedures varied by institution, but usually involved a person responsible for human research protection matters; federal government regulations required such a person in each institute. Private RECs in Canada and the USA nominated their own members.

In Finland, REC costs were covered by fees and public money, often from a special state subsidy intended to reimburse research costs in hospital districts. A handling fee was regulated by a sub-law and non-commercial researchers could apply for an exemption. In England, costs were fully covered by the NHS. In Canada, RECs charged industry but not academic researchers. Money came from the general funds of hospitals/health facilities or grant overheads. Likewise, in the USA, the costs were usually covered by institutions.

In England and the USA, RECs were regulated and supported by a public body (subsequently known as the REC control body) (Table 3). In Canada, there had been an educational body, the National Council on Ethics in Human Research, but its government funding was cut in 2010. In England, the REC control body (originally the Central office for RECs, and later the NRES) controlled local RECs, appointing, crediting and auditing them. The secretariats of RECs were NRES employees. NRES has been pro-active in its work, having reduced the number of RECs, streamlined and created common procedures, and introduced a central electronic submission system for applications. The NRES did not review applications and, in the case of a complaint, referred the application to another local committee for a second review.

In the USA, the OHRP registered RECs in response to applications and issued assurances of compliance with federal regulations. Researchers could complain to the OHRP, which could organize a re-review. The OHRP hosted a national committee (the Secretary’s Advisory Committee on Human Research Protections), which provided expert advice and recommendations to the health ministry on ethics and research regulation.

In Finland, the central committee (National Committee on Medical Research Ethics (TUKIJA)), appointed by the health ministry, had a dual function. It was the primary address for applications for multi-center drug trials (and for all drug trials since 2010). Secondly, it provided guidance and education to local RECs, giving second opinions in the case of complaints. However, the committee had no formal power over local RECs. Although the committee could delegate the handling of drug trial applications to local RECs, and most had been delegated, the emphasis was still on drug trial reviews and the committee’s other activities were modest. The central committee earned most of its income from handling fees.

## Discussion

Few previous studies have analytically compared research regulation between different countries. A study by Veerus et al. [7] compared the legislation on medical research and structural features between RECs in all EU countries. Two working reports have described differences and similarities in the regulation of drug trials in EU countries; one included England and Finland [8], while the other included England alone [9]. The study by Veerus et al. [7] was based on a synthesis of documents, while the two working reports were based on reports by country representatives, which varied from one country to another [8,9]. The facts available from the two reports of the two EU countries agree with the findings of this study. Veerus et al. [7] identified notable variation based on the type of medical research regulated by law, and on the numbers and working principles of RECs.

The overriding impression was that of a highly complex research regulation system, partly varying between different types of research and involving a range of actors. This agrees with previous conclusions based on evidence from England and the USA [6,10,11]. Protection of patient safety was the main aim of regulation, but many other interests were also involved, including the protection of economic, institutional, and professional interests. The aim of clinical research is to improve patient care, and the facilitation and regulation of clinical research should respond to that [1-3]. The current regulatory complexity appeared to be largely irrational, probably arising from piecemeal reactions to specific problems and scandals in the past. Thus, the new English HRA is of great interest in terms of future developments. If successful, it may have an impact outside England.

Various structures for regulating clinical research existed in all four study countries. Most structures could be found in all countries, but differed in their detail and scope, regardless of the international nature of biomedical ethics rules, medical research and health care content. Some such variation may be due to the government structure, as well as its organizing and financing of health care and medical research. Membership of the EU had an impact due to the influential clinical trials directive. Lacking data on the experiences of researchers and research subjects, it cannot be reliably judged which of the study countries had the best regulation system. However, if asked for a view, the English model could be examined for further ideas on reasonable research regulation in general, and the USA on drug research regulation in particular.

An important feature lay in the kind of research regulated within one (the main) system, i.e., whether this depended on the research type (Finland), the party funding the health system (England), or the research site (Canada, USA). In Finland, a clear distinction was made between medical and other human research. However, such a distinction was problematic, both theoretically and in practice, particularly given that medical research regulation was law-based while regulation of other research was voluntary.

Only Finland had specific and detailed legislation on medical research. However, all countries had a large number of legal and semi-legal rules regulating research and covering specific aspects of clinical research. Laws are a cumbersome tool for regulating research details, since legislation cannot account for all situations and exceptions. In this respect, the US’s approach of enacting a general research law to set principles, and regulating the details on the basis of rules which can be changed more easily, was better. The content of the detailed law in Finland may have contributed to clinical research regulation focusing on legality, patient safety, and scientific quality. Other aspects relating to good science (conflicts of interests, transparency, availability of results, etc.) were poorly developed.

The existence of various financial interests and pressures, including those of health service payers, may be a reason for the lack of a rational and simple regulatory framework for research. Research regulation is not a technical issue, but deals with power and money. It is a tool for determining whose view on medicine and health care will be followed. Much clinical research is related to the marketing of health care products. For drug firms, drug trials form part of the marketing chain [12]: drug trials are performed in order to obtain data for a marketing license, to tie experts and institutions into a product or firm, and to market the product before the sales license is granted. The complexity of research regulation had led to the formation of various large and small businesses with the aim of assisting researchers and sponsors [13] within contract research organizations [14]. The research business is likely to oppose simplifying and rationalizing the regulation system, since its livelihood depends on the system’s complexity. Conflicts of interest were an important regulation topic in Canada and the USA, but not in the two European countries studied.

Data on the costs of regulation systems was not available. However, the sheer volume of actors and of the requirements imposed on researchers suggests that the costs are high. Furthermore, the intimidating atmosphere created by some research regulation may have resulted in the assumption that certain activities are mandatory, even though this is not the case, e.g., in drug trial monitoring. Research costs have a direct impact on the volume of and approaches taken to research. In the long term, such costs are imbedded in the costs of health care. Previous studies on regulation costs as a whole were not found, but studies from the USA have estimated that the costs of one section of regulation, that imposed by RECs, were notable [15,16].

Clinical research with drugs was more extensively regulated than other clinical research, involving additional structures and specific regulatory bodies and laws. In all countries, the main tasks of the drug control authority (drug licensing and overseeing drug producers, sellers, and marketing) differed from those of the research world, which led to discrepancies. Extra regulation relating to drug trials may be due to history (visibility of drug related harms), the large volume of drug trials, and the strong financial power of the drug industry and its central role in drug research. Tighter control on drugs may be appropriate in the case of new products intended for routine clinical use. Legislation in the USA and Canada, restricting the mandate of drug control authority on research aimed at obtaining a marketing license, was better than that in Finland and England, in which a variety of poorly defined types of drug research were put under the same regulation. The main cause for this was the highly detailed EU Clinical trials directive, which covered all interventional drug research and the broad national interpretation of the term ‘interventional’. The various problems associated with the directive, and its failure to achieve the aims set for it, have been much discussed [1,8,17]. In 2014, the directive was revised to alleviate these problems, but it remains to be seen how the new law will change regulation in practice. However, there has been no change in the basic notion that drug research should have a more extensive and different set of regulations than other clinical research.

The role of health care proved difficult to define. Health care seemed to have various roles mixed with those of researchers, who were health care employees. In the USA and Canada, research regulation was related to the level of liability of health care units, which tended to make them avoid risk. Since clinical research has a direct impact on patient care, one might assume that health care has a strong interest in facilitating and directing clinical research. This was the case in England, but not in Finland; the data did not allow conclusions to be drawn about Canada or the USA with its highly varied health care system. In the USA, a topical issue was the integration of clinical trials and care [2]. The general understanding of what constitutes research and what requires informed consent seemed to be changing.

RECs were important to regulation in all four countries and their tasks included aspects other than ethics (Hemminki, unpublished manuscript 2014). Although RECs had similar core tasks, there were notable variations in the way in which RECs were organized and in discussions on the need to improve them. In England, REC activity had been transformed into a clear, and apparently well accepted, system. In Canada and the USA, RECs formed part of institutions (hospitals), which had the benefit of allowing closer integration of research with patient care. However, institutional conflicts of interest and institutional liability problems existed. Furthermore, it is likely that the large number of RECs led to variation and difficulties in multi-site research.

### Strengths and weaknesses

Most data from the four countries was collected by one person. Use of a single data collector was a strength which enabled the examination of each system from the outside on a similar basis. When local experts are asked to describe their own system, they commonly fail to observe the system’s particular features. Secondly, use of a single interviewer enabled information from one interview to be used in the next.

Weaknesses included the difficulties inherent in any country comparison, the limited amount of data collected on complex issues which were difficult to define, and the failure to define all aspects relevant to clinical research regulation equally well. As a result, the findings presented are simplifications. Only the main features were described, not the many exceptions and nuances. RECs were given the deepest coverage, since they were important actors and relatively comparable between countries; the content details will be reported elsewhere (Hemminki, unpublished manuscript 2015). No previous studies comparing all research regulation structures existed before the study, and several relevant aspects were only identified during data collection. As the interviews were aimed to gather basic descriptive information as well as the viewpoints of the informants, the aspects covered varied between interviews.

Inherent difficulties in country comparisons include those resulting from varying health care systems and roles played by government/public actors, and the fact that some tasks were handled by different actors in different countries. RECs and drug trial authorities were most comparable. Some of these structural differences became apparent only during the interviews. Due to limited resources, there was no way of approaching informants for a second time to enquire about items which had arisen after their interviews. A further difficulty was that, during the study, a great deal occurred in clinical research facilitation and regulation. Furthermore, the interviewed persons were usually aware of planned changes, which may have influenced their views accordingly.

Data confidentiality, data access, secrecy, and privacy issues were not systematically studied, even though they are topical to research regulation. Such issues are highly dependent on the general legislative framework and culture of each country, on which sufficient comparative data was lacking.

## Conclusions

This study demonstrated that a comparison between different countries can provide useful insights on the system within each country, as well as revealing common features which may require international action. In most cases, country specific differences probably do not correspond to actual differences in the need for research regulation. Differences in structure also suggest that the processes and end results can vary. My impression was that, while regulation was extensive, it is not necessarily focused on the correct issues. The complexity of the system suggests that useful research is not encouraged but may be hindered.

Certain features of research regulation in individual countries could serve as a model for other countries: the streamlining of the ethics committee system in England, the content advice provided on the handling of ethical issues in Canada, and the separation of drug trials to be used in licensing and other drug research in the USA are examples. Finland could learn a great deal from the other countries and an article has been written on improving the local control system [5].

Further country comparisons, with greater in-depth analysis of the key aspects, would be useful. Important aspects not covered are also worth studying, including the costs of the regulatory system and its outcomes: are research subjects being protected and is useful research being promoted?

## Abbreviations

CIHR: Canadian Institutes of Health Research
COI: Conflicts of interests
DHHS: Department of Health and Human Services
EU: European Union
FDA: Food and Drugs Administration
HRA: Health Research Authority
MHRA: Medicines and Healthcare Products Regulatory Agency
NHS: National Health Service
NIH: National Institutes of Health intramural and extramural funding
NIHR: National Institute of Health Research
NRES: National Research Ethics Service
OHRP: Office of Human Research Protection
RECs: Research ethics committees
